# Dynamics in C, N, and P stoichiometry and microbial biomass following soil depth and vegetation types in low mountain and hill region of China

**DOI:** 10.1038/s41598-021-99075-5

**Published:** 2021-10-04

**Authors:** Wenting Jiang, Lei Gong, Lihui Yang, Shuping He, Xiaohu Liu

**Affiliations:** 1 College of Life Science, Yan’an University, Yan’an, 716000 Shaanxi China; 2 College of Land and Environmental, Shenyang Agriculture University, Shenyang, 110866 China

**Keywords:** Forest ecology, Environmental chemistry

## Abstract

Changes in soil carbon (C):nitrogen (N):phosphorus (P) stoichiometry have great significance on understand regulatory mechanism and restoration of ecosystem functions. However, the responses of C, N and P stoichiometry to soil depth and different vegetation types remains elusive. To address this problem, the study aims to explore the effects of soil depth and vegetation types on soil C, N, and P stoichiometry, and their relationships with microbial biomass in low mountain and hill region of China. The results indicated that soil SOC and TN concentrations in oak forest were markedly higher than those in grassland, and the vertical distribution of SOC and TN concentration showed an inverted triangle trend as the soil deepens. However, there was no significant change in soil TP concentration among 0–20 cm, 20–40 cm, and 40–60 cm. Soil C/N among different layers (0–20, 20–40, and 40–60 cm) is narrower fluctuation margin, and its value is basically stable within a certain range (11–14.5). Both soil C/P and N/P showed significant variability in different vegetation types, and soil N/P decreased with soil layers deepen. Both the microbial biomass C (MBC) and N (MBN) showed a decreasing trend with the increase of soil depth, and three soil layers from high to low was: oak forest > pine forest > grassland. Our results will potentially provide useful information for the vegetation restoration and forest management and great significance to enrich the scientific theory of ecological stoichiometry.

## Introduction

In ecosystem, carbon (C), nitrogen (N) and phosphorus (P) as factors affecting biogeochemical cycling and important ecological processes, which play a key role in the formation and maintenance of substances^1–3^. Furthermore, the interaction of soil C, N, and P elements in the material cycle plays a crucial role in forest and grassland ecosystems productivity and carbon sequestration potential^4,5^, and have significant regulatory effects on soil erosion and subsequent deposition on biological activity. As dynamic components of the terrestrial ecosystem, soil organic carbon (SOC) are the major determinants and indicators of soil fertility and quality^6,7^. The largest pool of organic carbon is in the soil. Approximately the emission of 1500 Pg C in 1 m of the surface soil is twice as much as the carbon content in the atmosphere. The SOC pool emit about 1.6 Pg C annual year in the atmospheric carbon pool through tree cutting, wood burning, irrigation and drainage^8–10^. Therefore, Subtle changes in the SOC storage can lead to effects on the carbon dioxide atmospheric concentration and influence on changes in C cycling.

Numerous factors affect the cycling of soil nutrients in terrestrial ecosystems, and consequentially affect the distribution and stock of C and N^11,12^. One of the key factors is vegetation type change^13,14^. vegetation type change caused by human interference has been a common occurrence, and plays an vital effect on regulating nutrient levels in the terrestrial ecosystem. Different vegetation types may lead to different soil carbon (C) and nitrogen (N) input rates, and these SOC and TN changes will lead to the storage or loss of soil C and N^15^. Jobbagy et al.^16^ to evaluate the influence of different vegetation types on SOC based on analysis 2700 soil profiles in three global databases, and indicated that vegetation types significantly affected the vertical distribution of SOC. The percentage of SOC in the top 20 cm (relative to the first meter) averaged 33%, 42%, and 50% for shrublands, grasslands and forests. Similarly, Fu et al.^17^ conducted a researches from four major vegetation types of 12 slope land, and found that there were large variations in the distributions and stocks of SOC and N across the four vegetation types, and improvements of SOC and TN stocks in the transitional belt could be made through well vegetation management measures.

China is one of the rich forest and grass resource countries, mainly contains a large number of pine forest, oak forest, mixed forest, and grassland. This area belongs to the typical continental monsoon climate, with strong solar radiation, long sunshine duration and the same season of rain and heat, which is very conducive to the growth of forestry populations and plant dry matter accumulation. It has experienced remarkable changes in land use since the 1980s. However, long-term high-intensity utilization of land and unlimited exploitation of natural resources to meet ever-increasing demand of large population, thereby resulting in to soil erosion and sandstorm disasters occur frequently^18,19^. Compared with the non-cultivated soils, a reduction of 10–40% of SOC in cultivated soils had been reported in China with the highest losses experienced in the semi-arid and sub-humid areas^20^. In order to considering the benefits of soil quality improvement, the “Grain for Green” as the most effective way of environmental protection and soil ecological comprehensive treatment, considerable work has been performed by the Chinese government^21,22^. Meanwhile, these professional forest management models generate greater value in forest ecosystems. These forest type limited water and soil resources at the lowest cost, thereby improving the contaminated area of the soil^23,24^. Subsequently, a series of national conservation projects were launched in 1999, focusing on ecological restoration. The efforts are expected to help improve the regional eco-environment as well as the SOC sequestration, and many studies have investigated the these vegetation restoration strategies related to soil fertility^16–18^. However, information on the mechanism of the soil C, N, and P concentrations and stoichiometric ratios, and microbial biomass in different vegetation type and soil depth is still scarce in China, especially in low mountain and hill region. Thus, the field experiments were initiated to determined 0–60 cm soil profiles in three vegetation types of pine forest, oak forest, and grassland. The objectives of this study were to investigate variations in SOC, TN, and TP component, stoichiometry and their relationships, and further determine microbial biomass C (MBC) and N (MBN) following soil depth and vegetation types in low mountain and hill region.

## Results

### Vertical distribution characteristics of SOC, TN and TP concentrations in different soil depths and vegetation types

**Figure 1:**
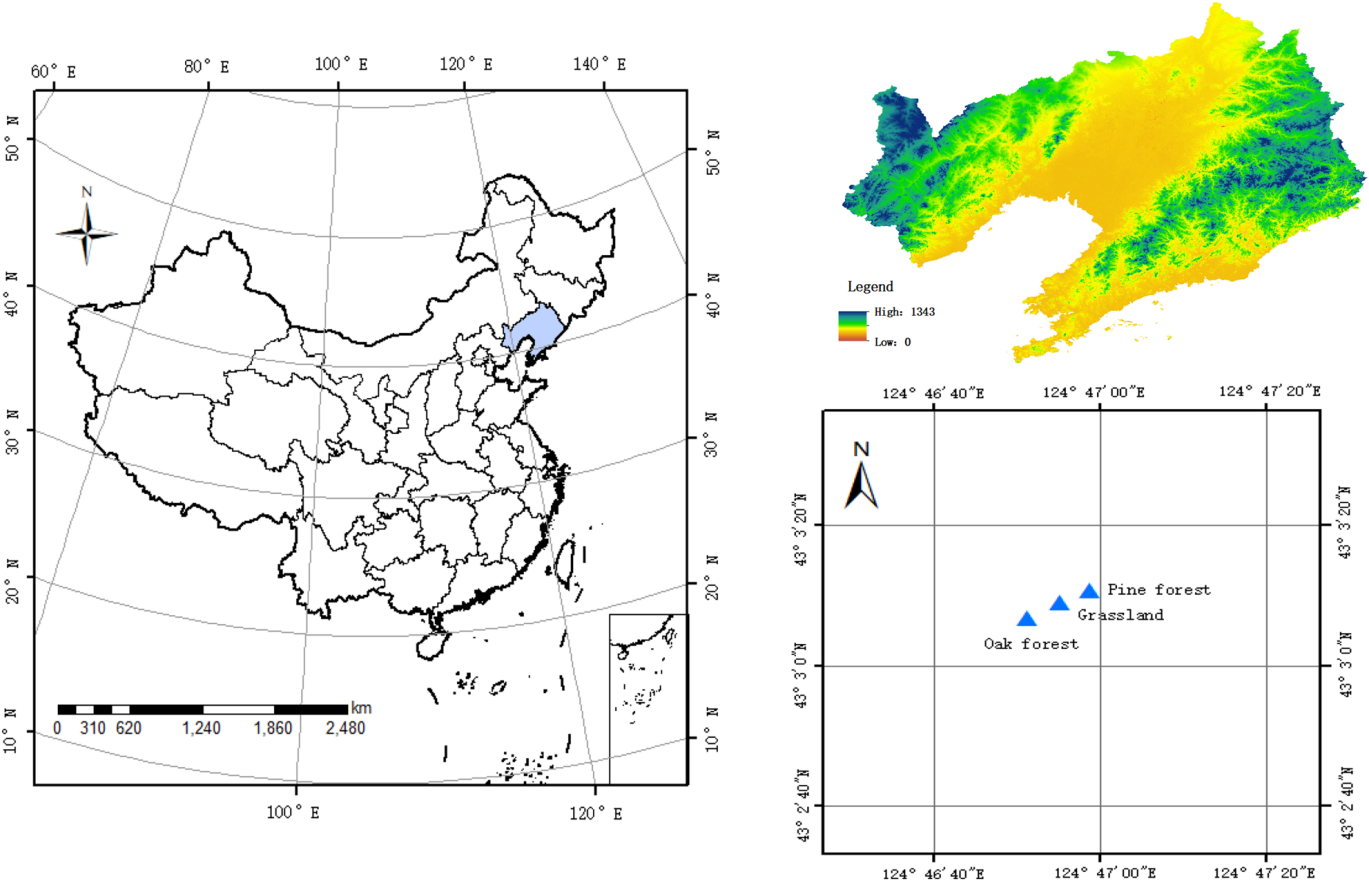
Distribution map of three experimental sites for different vegetation type (forestland, cropland, and abandoned land) in Liaoning province in northeast China. (Map created using ArcGIS 10.6 software by first author, URL: http://www.esri.com).

**Figure 2:**
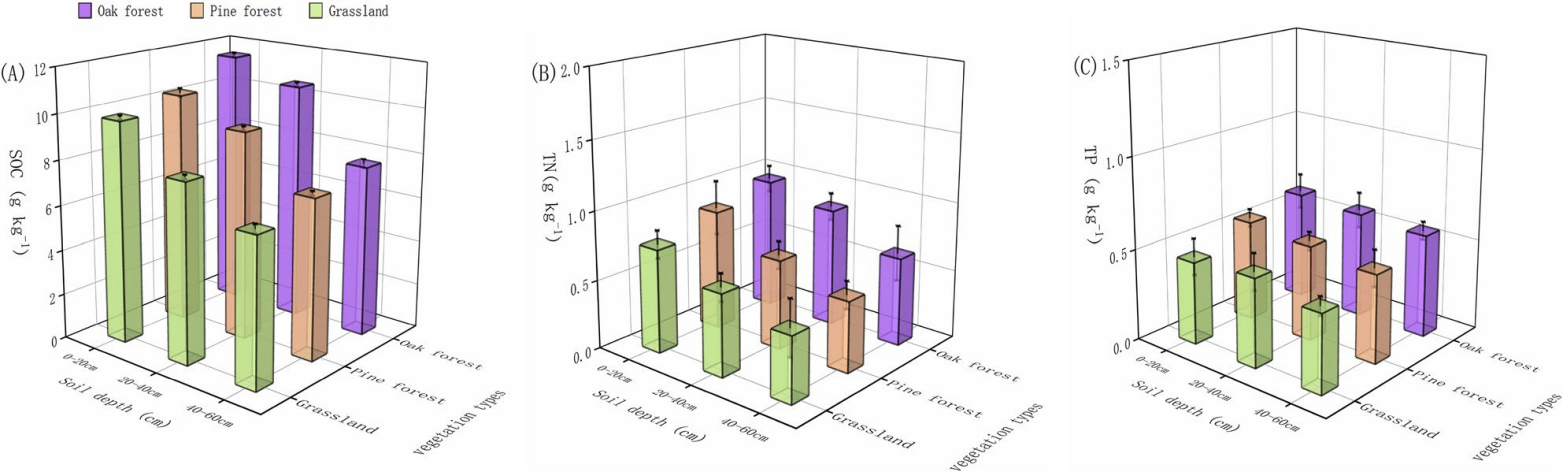
The SOC (**A**), TN (**B**) and TP (**C**) concentrations affected by vegetation types (oak forest, pine forest, and grassland) and soil depths of 0–20 cm, 20–40 cm, and 40–60 cm.

Three typical vegetation types including pine forest, oak forest, and grassland were selected used in our study (Fig. 1). The highest SOC concentration in 0–20 cm soil layer was measured in oak forest (11.5 g kg^−1^), 11.6% and 17.3% higher than that in pine forest and grassland, respectively (Fig. 2A). Soil Organic Carbon concentration of oak forest in 0–20 cm layer differed significantly (*p* < 0.05) from other vegetation types. In 20–40 cm, the lowest SOC concentration was measured in grassland (7.9 g kg^−1^) was significantly lower than that in other vegetation types (Fig. 2A). SOC concentration in 40–60 cm soil layer followed the same trend in 0–20 cm and 20–40 layers except that there was slightly difference in the amount of SOC among three vegetation types. It was remarkable that the SOC concentration in each vegetation types gradually decreased with the increase of soil layer, and the SOC concentration in 0–20, 20–40, and 40–60 cm layers from high to low was: oak forest > pine forest > grassland (Fig. 2A).

Concentration of soil total nitrogen (TN) followed a trend similar to that of SOC, gradually decreased with the increase of soil depth (Fig. 2B). In the 0–10, 10–20 and 20–40 cm soil layers, the average TN concentration was highest in oak forest, followed by that in pine forest and grassland. Accordingly, grassland in 0–20 cm layer with significant lowest TN (0.75 g kg^−1^), 21.8% and 13.7% lower than that in oak forest and pine forest, respectively (Fig. 2B). There were significant differences among the three vegetation types in the 0–20 cm soil layers. Soil TN concentration of oak forest in 20–40 cm layer had slightly 0.2 kg ha^−1^ and 0.27 g kg^−1^ higher than that in pine forest and grassland, respectively (Fig. 2B). The oak forest, pine forest and grassland in 0–20 cm layer showed similar trend in the 20–40 and 40–60 cm soil layers (Fig. 2B). Consistent with the concentration of SOC, the TN decreased with the increase of soil depth, and the highest TN in each soil layers was also in oak forest.

Under different vegetation types, the average concentration of total phosphorus (TP) in 0–60 cm soil layer was 0.45–0.6 g kg^−1^, and the concentration of TP in each soil depth had little difference among different vegetation types (Fig. 2C). Except for grassland, TP concentration in 0–20 cm soil layer in oak forest and pine forest was relative higher (Fig. 2C). There was no significant change in soil TP concentration among 0–20 cm, 20–40 cm, and 40–60 cm, and the concentration of TP goes lower as the soil deepens, but the decreasing trend is slow.

### Stoichiometric ratios of soil C, N and P

**Figure. 3:**
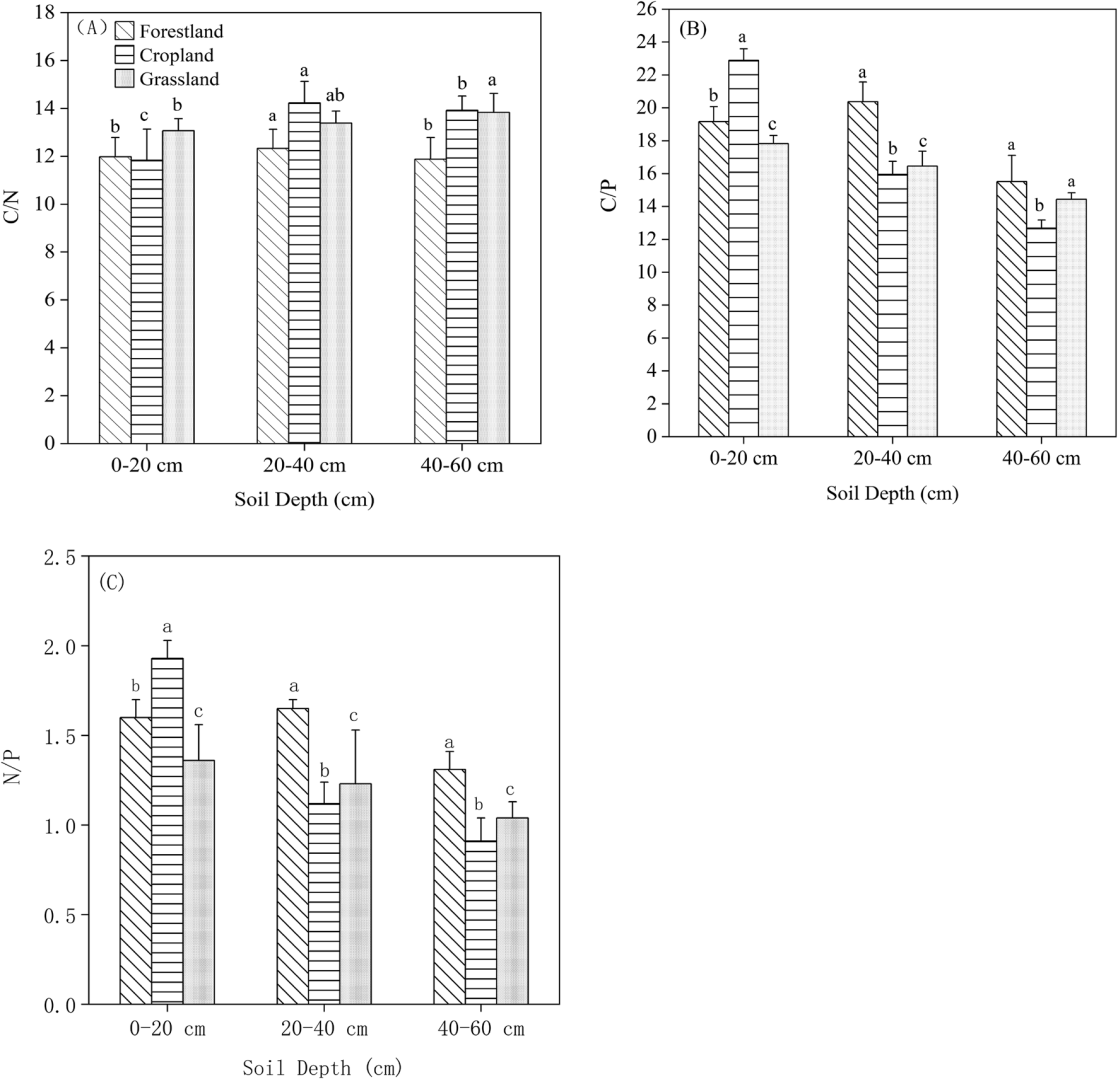
Variations in soil C/N (**A**), C/P (**B**), and N/P (**C**) stoichiometry ratios in different vegetation types and soil depths, respectively. The same letters indicate an insignifcance between different vegetation types at p < 0.05.

The soil C:N:P stoichiometry ratios is significant affected by different vegetation types and soil depths. The C/N range of 0–60 cm soil depth in all vegetation types ranged from 11.84 to 14.23, the highest C/N were measured in 20–40 cm soil layer of pine forest, and the lowest was measured in 0–20 cm soil layer of pine forest (Fig. 3A). In 0–20 cm, the soil C/N in grassland was 9.09% and 10.38% higher than that in oak forest and pine forest, and there were significant differences among oak forest, pine forest and grassland (Fig. 3A). The soil C/N of oak forest in 20–40 cm soil layer was 14.23, 15.4% and 6.27% higher than pine forest and grassland (Fig. 3A). In 40–60 cm soil layer, there are no significant differences in soil C/N between pine forest and grassland, and the lowest soil C/N was in oak forest. In oak forest and pine forest, the soil C/N increased until 40 cm soil layers and then decreased until 60 cm soil layer (Fig. 3A), and the C/N in grassland have no significant change from 0 to 60 cm.

Soil C/P is an important indicator of P availability. The C/P in all vegetation types decreased with soil layer deepen (Fig. 3B), ranging of 13.57–19.17 (oak forest), 14.79–18.73 (pine forest), 16.46–21.78 (abandoned land) from 0 to 60 cm soil layers, respectively. In 0–20 cm, the lowest soil C/P was in pine forest, 2.29% and 16.28% lower than that in oak forest and grassland. Different from 0 to 20 cm, the highest soil C/P in 20–40 cm was in oak forest, 2.75% and 11.05% higher than that in pine forest and grassland (Fig. 3B). There are significant differences in 40–60 cm soil layer among oak forest and pine forest, and grassland.

Soil N/P followed a trend that gradually decreased with the increase of soil depth. Remarkably, the soil N/P in pine forest in 0–20 cm layer was 1.58, which were 1.25% and 5.3% lower than that in oak forest and grassland, respectively (Fig. 3C). In 20–40 cm layer, soil N/P in oak forest had 18.4% and 1.62% higher than that in pine forest and grassland, respectively. Pine forest in 40–60 cm soil layer with lowest soil N/P (1.06), which were 7.01% and 5.35% slightly lower than that in oak forest and grassland, respectively (Fig. 3C). There were no significant differences among the three soil utilization patterns in the 40–60 cm soil layers.

### Relationship among SOC, TN, TP concentrations and C/N, C/P, N/P stoichiometry

**Figure 4:**
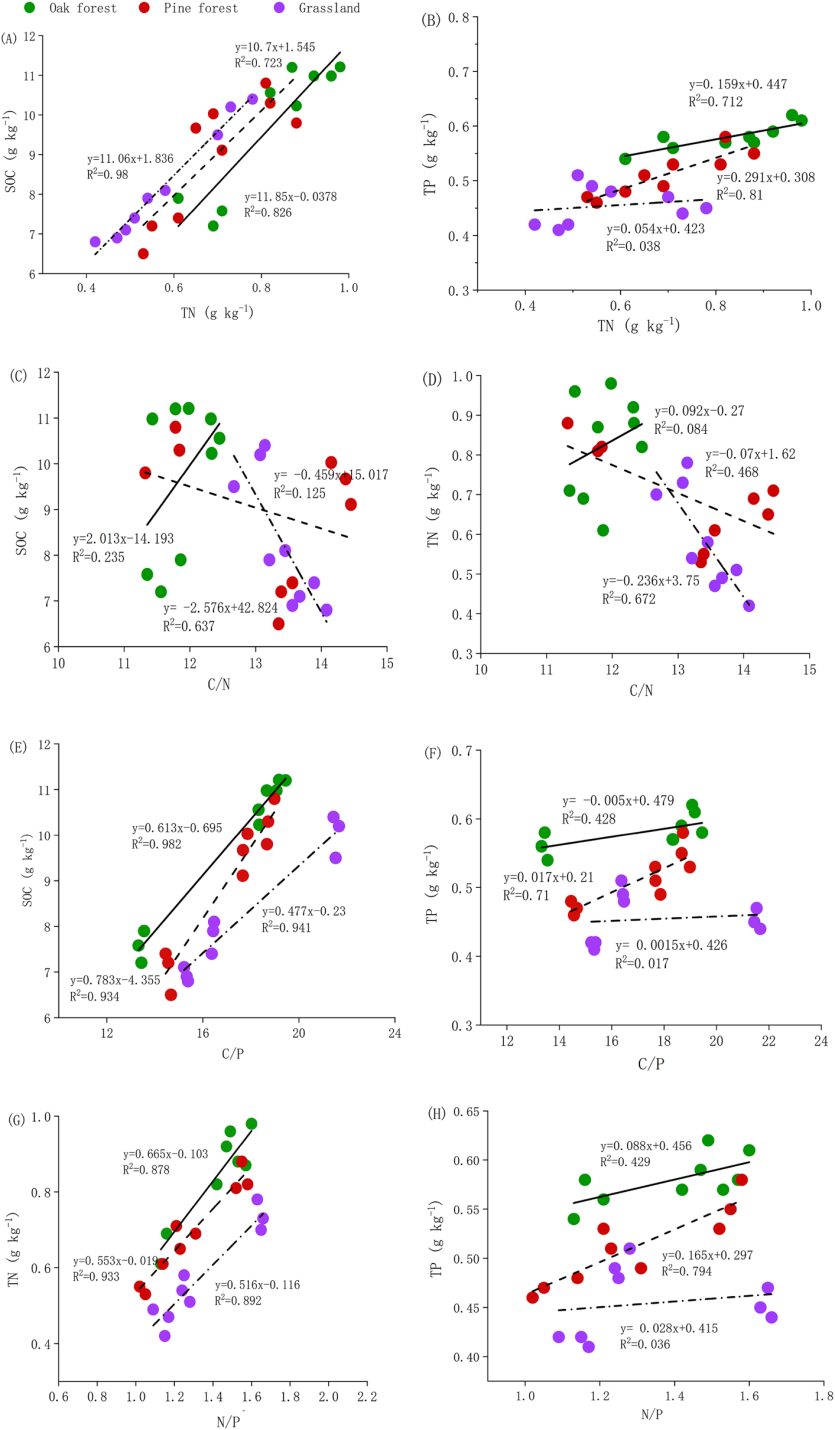
Relationships between TN and SOC (A), TN and TP (**B**), C/N and SOC (**C**), C/N and TN (**D**), C/P and SOC (**E**), C/P and TP (**F**), N/P and TN (**G**), N/P and TP (**H**) stoichiometry ratios, respectively (p < 0.05).

There was a certain correlation between the concentrations of three indicators SOC, TN, and TP, but the significant levels were different (Table 1). There were significant linear correlations between TN and SOC in three vegetation types, including oak forest (*R*^2^ = 0.826), pine forest (*R*^2^ = 0.723), and grassland (*R*^2^ = 0.98) (Table 1 and Fig. 4A). Similarly, There was a significant linear function relationship between TN and TP in oak forest (*R*^2^ = 0.712) and pine forest (*R*^2^ = 0.81), and except for grassland (*R*^2^ = 0.038) (Fig. 4B).

**Table 1:** Correlation analysis of soil SOC. TN, TP and stoichiometry ratios in different vegetation types (*p* < 0.05).

x	Vegetation types	y	
		SOC	TP
TN	Oak forest	y = 11.85x − 0.378 *R*^2^ = 0.826	*y* = 0.159*x* + 0.447 *R*^2^ = 0.712
	Pine forest	y = 10.7x + 1.545 *R*^2^ = 0.723	*y* = 0.291*x* + 0.308 *R*^2^ = 0.81
	Grassland	y = 11.06x + 1.836 *R*^2^ = 0.98	*y* = 0.054*x* + 0.423 *R*^2^ = 0.038

x	Vegetation types	y	
		SOC	TN
C/N	Oak forest	*y* = 2.013*x* − 14.192 *R*^2^ = 0.235	*y* = 0.092*x* − 0.27 *R*^2^ = 0.084
	Pine forest	*y* = − 0.459*x* + 15.027 *R*^2^ = 0.125	*y* = − 0.07*x* + 1.62 *R*^2^ = 0.468
	Grassland	*y* = − 2.576*x* + 42.82 *R*^2^ = 0.637	*y* = − 0.236*x* + 3.75 *R*^2^ = 0.672

x	Vegetation types	y	
		SOC	TP
C/P	Oak forest	*y* = 0.613*x* − 0.695 *R*^2^ = 0.982	*y* = − 0.005*x* + 0.479 *R*^2^ = 0.428
	Pine forest	*y* = 0.783*x* − 4.355 *R*^2^ = 0.934	*y* = 0.017*x* + 0.21 *R*^2^ = 0.71
	Grassland	*y* = 0.477*x* − 0.23 *R*^2^ = 0.941	*y* = 0.0015*x* + 0.426 *R*^2^ = 0.017

x	Vegetation types	y	
		TN	TP
N/P	Oak forest	*y* = 0.665*x* − 0.103 *R*^2^ = 0.878	*y* = 0.088*x* + 0.456 *R*^2^ = 0.429
	Pine forest	*y* = 0.553*x* − 0.019 *R*^2^ = 0.933	*y* = 0.165*x* + 0.297 *R*^2^ = 0.794
	Grassland	*y* = 0.516*x* − 0.116 *R*^2^ = 0.892	*y* = 0.028*x* + 0.415 *R*^2^ = 0.036

There was no significant relationship between C/N and SOC (*R*^2^ = 0.235, 0.125, and 0.637) (Fig. 4C), and between C/N and TN (*R*^2^ = 0.084, 0.468, and 0.672) (Fig. 4D) in three vegetation types. The relationship between C/P and SOC showed a significant linear correlation in oak forest (*R*^2^ = 0.818), pine forest (*R*^2^ = 0.934), and grassland (*R*^2^ = 0.941) (Fig. 4E), whereas no significant relationship was found with C/P and TP (Table 1, Fig. 4F). The N/P was significantly positively linear correlated with the TN (oak forest *R*^2^ = 0.878, pine forest *R*^2^ = 0.933, and grassland R^2^ = 0.697) (Fig. 4G), but no significant correlation was found with N/P and TP (Fig. 4H).

### Differences of soil microbial biomass carbon (MBC) and nitrogen (MBN) in different vegetation types

**Figure 5:**
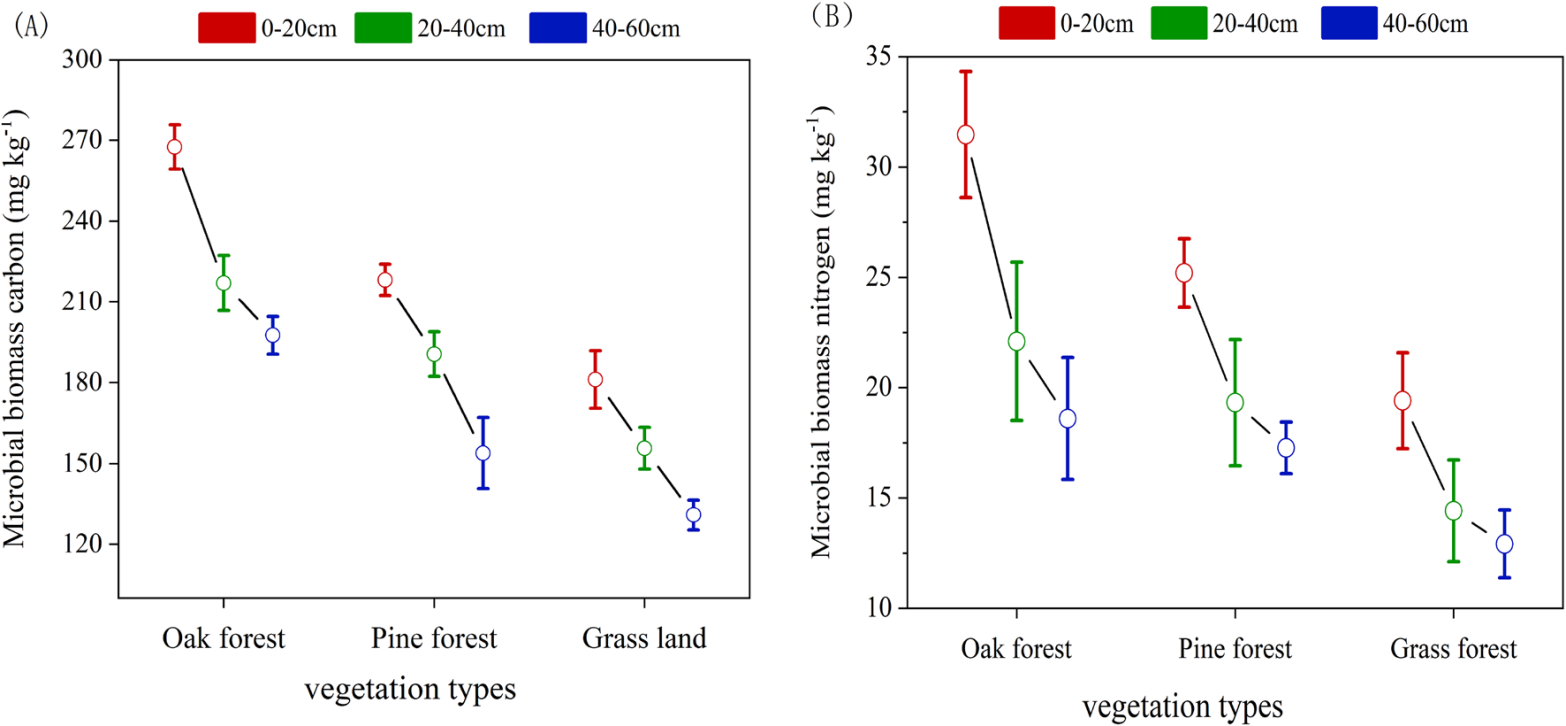
Variations in MBC and MBN affected by different vegetation types and soil depths.

There were significant differences in the MBC at three vegetation types at each soil depth (0–20 cm, 20–40 cm and 40–60 cm). In the 0–20 cm soil depth, the soil MBC concentrations in oak forest were significantly higher than that in pine forest and grassland (Fig. 5). The soil MBC concentration at the 0–20 cm soil depth decreased from 267.8 mg kg^−1^ in the oak forest to 185.4 mg kg^−1^ at the 40–60 cm. Similarly, the soil MBC concentrations in pine forest and grassland were gradually decreased with the increase of soil depth. The lowest concentrations of MBN were observed in grassland at both the 0–20 cm, 20–40 cm and 40–60 cm soil depths, which were 19.8 mg kg^−1^, 14.5 mg kg^−1^, and 12.7 mg kg^−1^ (Fig. 5). However, the soil MBN concentration in the oak forest was 22.56% and 59% higher than that in the other two vegetation types in 0–20 cm soil layer (Fig. 5). In the 20–40 cm and 40–60 soil layer, the soil MBN concentrations in three vegetation types were significant difference, and both of pine forest and oak forest were lower than the soil MBC concentration in oak forest (p < 0.05) (Fig. 5). Both the MBC and MBN showed a decreasing trend with the increase of soil depth, and three soil layers from high to low was: oak forest > pine forest > grassland.

## Discussion

### Soil organic carbon (SOC), total nitrogen (TN), total phosphorus (TP) concentrations in different vegetation types and soil depths

The soil elements of C, N and P can effectively affect plant growth, development and important chemical elements in the process of material cycling. Different vegetation types have a profound impact on SOC and TN concentrations^25^. In all vegetation types, the SOC content in 0–20 cm soil layer was much higher than that in other soil layers, and indicated that the vertical distribution of SOC and TN concentration showed an "inverted triangle" trend from 0 to 60 cm soil layers. The SOC and TN concentrations showed a trend of gradually decline with the soil layer deepening, which was consistent with the research results of Fu et al.^26^. The reason is that the aeration ability of the upper soil is better than that of the deeper soil, and nutrients are transported from the upper to the deeper layer, the accumulation of SOC and TN will first gather in the upper soil, and therefore the SOC and TN concentrations in the surface soil is significantly higher than that in deeper soil^27,28^.

Although the SOC for all three vegetation types were relatively small, the vegetation types (oak forest, pine forest and grassland) still significant affected SOC distributions and stocks in varying degrees in our study. The order of soil SOC and TN concentrations from low to high was oak forest, pine forest and grassland. The reason is that SOC concentration is mainly affected by plant litter, microbial residue and root exudates. Wang et al.^20^ proved that litter biomass and live biomass were closely related to SOC and STN, respectively. In the process of litter decomposition, carbon transport is a release process, while nitrogen transport is not only a release process, but also a concentration process^29^. Thus, the amount of soil plant residues and dead leaves under forest cover is relative large. In particularly, nutrients will be released into the soil during the process of the decomposition of root exudates, and increases the concentrations of SOC and TN in oak forest and pine forest, which is significantly higher than that in grassland. This is in agreement with Albrecht and Kandji^30^, who reported that adequate management of forestlands, a large fraction of the atmospheric CO_2_ could be trapped and stored both in forest biomass and soils. Takimoto et al.^31^ study the carbon sequestration potentials in West African Sahel indicated that a large proportion of the total amount of carbon in the agroforestry system is stored in the soil. In addition, the small amounts of litter biomass in pine forest compared with that of oak forest, is an important reason for smaller SOC and TN concentrations.

The vertical change of TP concentration was different from that of SOC and N concentrations, and there is no significantly change of soil P content with the increasing soil depth. Zhang et al.^32^ also reported similar conclusions on the TN content of different vegetation types in the loess hilly region. The reason is that phosphorus as a sedimentary mineral, which is mainly affected by soil parent material, soil formation and climate, and difficult to migration^15^. Thus, there is no significant difference in soil TP concentration among the three different vegetation types. Remarkable, the TP concentration in oak forest and pine forest was slightly higher than that of grassland, and the reason that the natural forest have rich in litter, which conducive to the accumulation of phosphorus.

### The C:N:P stoichiometric ratio in in different vegetation types and soil depths

Soil stoichiometric ratio of C, N and P used as a key performance indicator to reflect soil quality and composition. C/N is an index to measure the nutrient balance of soil C and N, and is often used as a sensitivity index to judge the change of soil quality. The results of this study showed that soil C/N among different layers (0–20, 20–40, and 40–60 cm) is narrower fluctuation margin, and its value is basically stable within a certain range (11.84–14.23), which is consistent with the results of other studies. Soil C/P is an indicator of measure to P release from microbial mineralized soil, has an important effect on plant growth and development. In our study, the soil C/P in all vegetation types decreased with soil layer deepen, and even which declined much faster than the C/N ratio. This results agreement with Tian et al.^33^ and Walker and Adams^34^. This is mainly because of the soil TP concentration was relative stable throughout the soil layers when compared to the rapid decline in SOC with soil depth (Fig. 3). Thus, we found that despite large variations in SOC and TN concentration, low soil TP concentration always caused by high C/P and N/P. In our current study, the value of soil C/P for all vegetation types and soil depths in the study area was ranged from 13.57 to 21.78, much lower than the average value of land in China^35^, similar to the soil C/P of Black Locust in Loess Plateau (8.66–25.47)^36^. Low C/P is an indicator of high soil P availability. Thus, our results showed that the soil P availability in our study area was relatively high, and the mineralization rate of P was relatively high, which was consistent with the research results of Gao et al.^35^. Soil N/P values as diagnostic indicators for N saturation and used for determining nutrient limitation thresholds. In this study, the mean N/P value was 1.56, which lower than the average value of 3.9 in China, and further verified that the severe lack of N in our study region.

### Effects of vegetation types on soil microbial biomass C and N

Soil microbial biomass plays an important role in indicating soil fertility, which is easily affected by forest litter, root exudates, fertilization, tillage measures and the addition of exogenous organic materials^37^. This study showed that the soil microbial biomass carbon and nitrogen of oak forest and pine forest were significantly higher than those of and grassland, which mainly due to the difference of stable organic matter input into soil by different vegetation types. These results consistent with the Jiang et al.^38^ results. There have abundant roots and large aboveground biomass in forestland, and provide a mass of litter for soil microorganisms and become natural organic fertilizer, and improve the nutrient status of soil biomass^39^. Meanwhile, root exudates and rotting roots can also provide abundant energy for microorganisms, and therefore the microbial biomass of oak forest and pine forest is the higher in carbon and nitrogen. This indicated that forestland was more beneficial to accumulate soil microbial biomass carbon and nitrogen in the study area, which agreement with the research results of Devi et al.^40^.

The soil MBC and MBN in grassland is mainly affected by the litter decomposition, especially natural grassland itself with abundant root exudates, which makes grassland not only has a large above-ground biomass, but also can provide a large amount of litter for soil microorganisms and become natural organic fertilizer, and thus organic fertilizer provides nutrients for microbial activities for grassland, thus promote the mass reproduction of microorganisms and increased the MBC and MBN. Furthermore, a large amount of root exudates and decaying fibril can also provide abundant energy for microorganisms. Although the above-ground biomass of the grassland is high each year, but the grassland affected by management factor (grazed, irrigation, mow). The above-ground grass is artificially removed, resulted in very little organic matter being introduced into the soil and the microorganisms lack of energy, and caused soil MBC and MBN concentration were decreased in grassland^41^. The difference of soil MBC/MBN in different soil patterns is mainly caused by the decomposition of vegetation litters and root, which forms different microbial communities. Vegetation can also change the microbial composition in soil by influencing soil moisture and soil nutrients. Different the amount of soil nutrients in different vegetation types lead to a great impact on soil MBC and MBN.

Moreover, the vertical distribution characteristics of soil MBC and MBN will affect the growth of aboveground plants. Our research showed that the soil MBC and MBN of three vegetation types decreased by the soil layers deepen, which indicated that soil MBC and MBN of soil surface in three vegetation types were higher than that in the lower soil layers. This results mainly because of SOC and TN in the soil surface is higher, and good air permeability is conducive to the growth of the soil microbial breeding. Soil microorganisms provide nutrients for plants through the process of decomposition of organic matter, which can effectively avoid ineffective nutrient loss and caused changes in soil microbial biomass^42–44^. It is concluded that soil MBC are closely related to soil carbon storage, which is consistent with the results of Li et al.^42^.

## Methods

### Site description

The study site was located in Xifeng county, Liaoning Province of Northeast China. Geographically, the study site is located between 43.06°N latitude and 124.77°E longitude, having an elevation from 267 to 445 m above sea level, average radiation intensity for monthly was 502.2 MI/m^2^. The relative humidity was 63%. This region has a typical subtropical monsoon climate with frost-free period between 140 and 190 days. The mean annual precipitation of 618 mm, nearly 70% rainfall occurs from May to October, with a large inter-annual variation. The selected sites were representative of the regional vegetation types in northeast China. The land forms are characterized by low mountains and hills, with the soil type is classified as brown soil (Keys to Chinese Soil Taxonomy) or classified as Haplic-Udic Luvisols according to the FAO soil classification.

### Soil sampling and preparation

According to the different vegetation types of the study region, three typical vegetation types including pine forest, oak forest, and grassland were selected in May 2018 (Fig. 1). Five sampling points were set in the shape of "S" in each vegetation types plots, with three sampling plots (20 m × 20 m, 400 m^2^) were set up. Soil samples in each vegetation-type area were taken from 0 to 20 cm, 20 to 40 cm, and 40 to 60 cm depths using a soil auger with a 5 cm diameter. In all, 27 soil samples (3 vegetation types × 3 sampling sites × 3 soil depths) were collected, and stored in cloth bags were air-dried at room temperature to constant weight. Soil samples are screened for impurities, such as decomposed plant roots, leaves and pebbles, and stored in the laboratory for analysis.

### Sample analysis and determination

The soil sample is divided into five parts, and used to measure soil organic carbon (SOC), total nitrogen (TN), total phosphorus (TP), Soil microbial biomass carbon (MBC), and microbial biomass nitrogen (MBN) concentration. The air-dried soil samples being passed through a 2 mm sieve before performing the nutrient analysis. The TN concentration was measured by the Kjeldahl digestion procedure, and the TP concentration was determined using molybdenum antimony blue colorimetry method^45,46^. The MBC concentration was extracted by chloroform fumigation and leaching, and MBN concentration was extracted by chloroform fumigation and leaching with ninhydrin colorimetric method^47^.

### Statistical analysis

The ANOVA procedure and Duncan test were analyzed the effects of different soil depth and vegetation types were subjected to SPSS19.0 software for Windows for statistical analyses. All results were shown as mean value of three replicates with standard errors. Least significant difference (LSD) method was used for multiple comparisons. Standard deviations were calculated for mean values of all the determination. Base map and data obtained from OpenStreetMap and OpenStreetMap Foundation (http://www.openstreetmap.org).
